# Insights into Human Ciliopathies: Gene Silencing of *ropn1l* and *tex9* in *Schmidtea* *mediterranea* Indicate Association with Ciliary Structure and Motility

**DOI:** 10.17912/micropub.biology.002035

**Published:** 2026-04-16

**Authors:** Rachel Pitt, Chayanika Gogoi, Angelina Calnan, Lata Patnaik, Kristen C Johnson

**Affiliations:** 1 Department of Life Sciences, University of New Hampshire at Manchester, Manchester, New Hampshire, United States

## Abstract

Cilia are microtubule-based organelles essential for motility, sensory signaling and development. In humans, motile cilia facilitate fluid movement, and their dysfunction causes ciliopathies, including infertility. We used RNAi-mediated knockdown of two human spermatid flagellar genes in *Schmidtea mediterranea* to assess effects on ciliary function and locomotion. Knockdown of *Smed-ropn1l* and *Smed-tex9* significantly reduced planarian swim rate by 26.4% and 33.2% respectively and shortened cilia by 37.1% and 38.7% respectively. These findings highlight the critical roles of ROPN1L and TEX9 in cilia function and the use of planarians as a valuable model for studying ciliopathies.

&nbsp;

**Figure 1. Knockdown of Smed-ropn1l and Smed-tex9 reduces planarian gliding locomotion and cilia length:** **A) Pathway tracing of RNAi-mediated knockdown worms shows reduced gliding motility.** Overlays of sequential frames from 20 second video segments, illustrating the distance travelled by individual flatworms in control ( *neg.ctrl.(RNAi)* ), *ropn1l(RNAi),* and *tex9(RNAi)* worms. Z-projection within FIJI/Image J software was used to perform the path tracing analysis. Scale bar = 10mm&nbsp;&nbsp; **B) Differential Interference Contrast Microscopy images at 63X magnification show planarian cilia length.** Gene knockdown of *ropn1l* and *tex9* resulted in visibly shorter cilia. Scale bar = 10µm. **C) RNAi-mediated knockdown impairs planarian swim speed.** Mean swim speed (± SEM) for *neg.ctrl.(RNAi)* worms was 1.3 ± 0.05 mm/s. *ropn1l(RNAi)* and *tex9(RNAi)* worms displayed 0.96 ± 0.17 mm/s and 0.87 ± 0.09 mm/s swim speeds, respectively (****p<0.0001, one-way ANOVA, n=30).&nbsp; **D) RNAi-mediated knockdown reduces cilia length in planarians.** Mean cilia length (± SEM) in *neg.ctrl.(RNAi)* worms was 11.04 ± 0.19 µm. *ropn1l(RNAi)* and *tex9(RNAi)* worms exhibited 6.94 ± 0.40 µm and 6.77 ± 0.30 µm cilia lengths, respectively (****p<0.0001, one-way ANOVA, n=5). &nbsp; **E) Single-cell RNA-Seq expression of *ropn1l* (dd_Smed_v6_4888_0) in planarian cell clusters.** Expression of *ropn1l* is strongly enriched in pharyngeal, epidermal, and specific neuronal cells with lower expression in other lineages (lineage diagram created using Planaria Single Cell Database; https://shiny.mdc-berlin.de/psca/). **F) Single-cell RNA-Seq expression of *tex9* (dd_Smed_v6_7007_0) in planarian cell clusters.** Expression of *tex9* is strongly enriched in pharyngeal and neuronal cells with lower expression in other lineages (lineage diagram created using Planaria Single Cell Database; https://shiny.mdc-berlin.de/psca/).

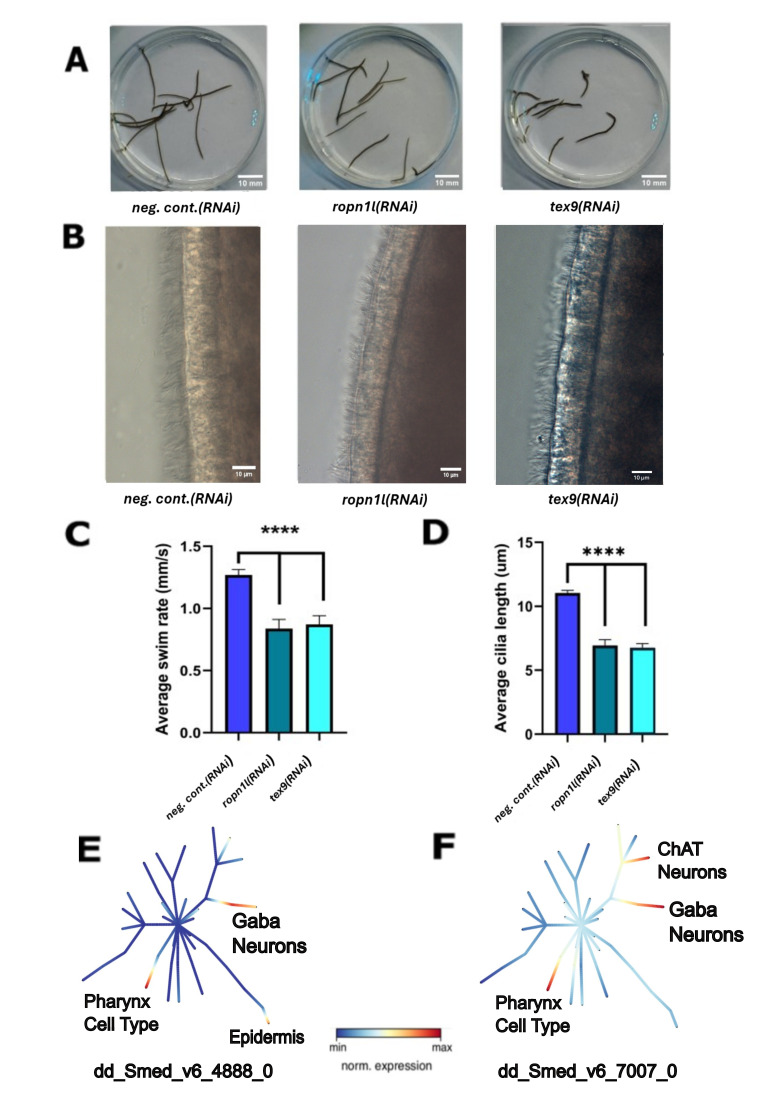



## Description

Cilia are small hair-like structures that are highly evolutionarily conserved and found on the surface of most eukaryotic cells (Horani & Ferkol, 2021). There are two main types of cilia, primary (non-motile) and motile which include flagella and nodal cilia (Hyland & Brody, 2021). Motile cilia and related microtubule-based structures, such as flagella, are essential for a wide range of cellular and developmental processes across eukaryotes. Ciliopathies are a set of human diseases arising from genetic mutations that affect cilia structure or function. Many ciliopathies currently lack curative treatments, although ongoing research is exploring gene therapy approaches that aim to address underlying causes rather than only managing symptoms (Wee et al., 2024). Yet, our understanding of these diseases is still not fully understood (Hyland & Brody, 2021).

Researchers have identified 1,999 unique ciliary genes, accounting for approximately 10% of all known human genes (Van Sciver & Caspary, 2024). Despite their importance, however, many cilia-associated proteins remain poorly characterized. Rhophilin associated tail protein 1–like (ROPN1L) and testis-expressed protein 9 (TEX9) have been identified through proteomic and transcriptomic studies as candidate components of cilia or flagella in humans, mice, and flies, with some evidence suggesting roles in flagellar or sperm tail structure. However, direct functional analysis of these proteins is limited. To address this gap, we used RNA interference to examine the functions of *Smed-ropn1l* and *Smed-tex9* , planarian homologs of the human genes, in *Schmidtea mediterranea* , a model well suited for studying motile cilia, and evaluated their roles in ciliary structure and function.

ROPN1L is a scaffold protein in the ROPN1/ROPN1L–Dependent 2 (R2D2) family that anchors protein kinase A (PKA) signaling complexes to cilia and flagella (Bontems et al., 2014). In human bronchial epithelial cells, *ROPN1L* is identified as one of 37 key cilia-associated genes upregulated during mucociliary differentiation, based on transcriptomic analysis (Ross et al., 2007). Proteomic and immunolocalization data from the same study further demonstrates that ROPN1L localizes to the ciliary axoneme, providing direct evidence of its association with cilia (Ross et al., 2007). Functional evidence for the role of ROPN1L in microtubule-based motility comes from mouse models, where knockout of *Ropn1l* or its paralog rhophilin associated tail protein 1 ( *Ropn1* ) results in moderately reduced sperm motility, while deletion of both genes causes complete sperm immotility, structural disruption of the fibrous sheath, and loss of A kinase anchoring protein 3 (AKAP3) in sperm flagella (Fiedler et al., 2013). Consistent with these findings, qPCR and RNA-seq analyses of germ cells from men with cryptorchidism, a congenital condition in which one or both testes fail to descend into the scrotum, reveals *ROPN1L* as the most downregulated gene among several fertility-associated targets, including *AKAP4, IZUMO1,* and *SPAG6,* further supporting its role in human fertility (Sun et al., 2023).

Functional and comparative genomic analysis of TEX9 across eukaryotes suggests a role for TEX9 in cilia biology. Phylogenetic profiling across 100 eukaryotic genomes identifies TEX9 as a candidate ciliary protein, and cross-species comparisons of flagellated and non-flagellated organisms places TEX9 within a highly conserved subset of six proteins associated with cilia-related centriolar satellites (Wu et al., 2025). Consistent with these predictions, CilioGenics—an integrative platform combining single-cell RNA sequencing, protein–protein interactions, comparative genomics, transcription factor network analysis, and text mining—identifies *TEX9* among 256 candidate ciliary genes, including 89 previously annotated by CiliaCarta and 28 with experimental validation (Pir et al., 2024). TEX9 is detected in human testes via proteomic analysis, suggesting an association with sperm development and cilia or flagella formation (Vandenbrouck et al., 2020). Direct evidence for ciliary association is provided by immunofluorescence studies demonstrating localization of TEX9 to ciliary basal bodies in cultured human kidney proximal tubule epithelial (HK2) cells (Nevers et al., 2017). Functional support for a role in cilia and flagella comes from loss-of-function studies, where RNAi knockdown of *TEX9* in *Drosophila* causes sensory cilia defects and impaired sperm flagellar motility, and *C. elegans* mutants exhibit dye-fill defects indicative of structural or trafficking abnormalities in sensory cilia (Dobbelaere et al., 2023).

Widely studied for its regenerative abilities, *S. mediterranea* (freshwater planaria) has a fully sequenced genome and robust molecular tools, including RNAi for gene function analysis, making it an ideal model for studying ciliary genes. The planarian is a low-cost, easily maintained laboratory model and is one of few multicellular organisms with an external multiciliated epithelium. Cilia are used in planarian locomotion and are external and visible in live specimens under the microscope (Rabiasz & Ziętkiewicz, 2023; Rompolas et al., 2009; Rompolas et al., 2010). Ventral epithelial cells bear multiple cilia that beat against a secreted mucus layer, together with muscle contraction, to drive locomotion, similar to mucus transport in the human airway, reproductive tract, and brain ventricles. Defects in ciliary function cause visibly impaired locomotion (Rompolas et al, 2010). Additionally, protonephridia depend on ciliary movement for waste filtration and osmoregulation, and ciliary disruption can impair this function, resulting in fluid retention and edema. (Reddien et al., 2005; Rink et al., 2009; Rink et al., 2011; Thi-Kim Vu et al., 2015).

RNAi-mediated knockdown of *ropn1l* and *tex9* in *S. mediterranea* resulted in clear phenotypic changes in swim rate when compared to *neg.cont.(RNAi)* ( Fig. 1A ; Extended data video 1 – *neg.cont.(RNAi)* 20 sec., Extended data video 2 – *ropn1l(RNAi)* 20 sec., Extended data video 3 – *tex9(RNAi)* 20 sec.). One week post-RNAi treatment, gliding locomotion was assessed using 20 second video segments, and distance traveled over time was determined using z-projection in Fiji/Image J. Quantitative analysis (n = 30) demonstrated that control worms ( *neg.cont.(RNAi))* had a mean swim rate ± SEM of 1.30 ± 0.05 mm/s, whereas *ropn1l(RNAi)* and *tex9(RNAi)* worms had significantly reduced swim speeds. *ropn1l(RNAi)* mean swim rate was 0.96 ± 0.17 mm/s and *tex9(RNAi)* mean swim rate was 0.87 ± 0.09 mm/s, representing decreases of 26.4% and 33.2%, respectively. (Fig 1C).

Differential interference contrast microscopy images of cilia located in the lateral region of planarian heads were taken, and these indicated reduced cilia length for *ropn1l(RNAi)* and *tex9(RNAi)* treated worms (Fig 1B). Quantitative analysis demonstrated significant reductions in cilia length in *ropn1l(RNAi)* and *tex9(RNAi)* worms, corresponding to decreases of 37.1% and 38.7%, respectively (mean ± SEM: 6.94 ± 0.40 µm and 6.77 ± 0.30 µm), relative to negative control ( *neg.cont.(RNAi)* ) worms (11.04 ± 0.19 µm) (Fig 1D). These data strongly suggest that functionally, these genes are important for ciliary integrity. In addition, edema in the trunk region of a small number of the *ropn1l(RNAi)* and *tex9(RNAi)* worms was observed, indicating potential defects in fluid regulation by the flame cells of the protonephridia (Reddien et al.,2005; Rink et al., 2009; Rink et al., 2011; Thi-Kim Vu et al., 2015). This edema was not observed in any of our *neg.cont.(RNAi)* worms (Extended data image 1 – edema&nbsp;observations).

Single-cell transcriptomic analysis of *ropn1l* and *tex9* obtained from Planaria Single Cell Database ( https://shiny.mdc-berlin.de/psca/ ) reveals that these genes exhibit strongest expression in specific neuronal subtypes, epidermal populations, protonephridia, and a distinct pharyngeal cell type described as epidermal-related ( Fig. 1E, F) (Plass et al., 2018). In addition, single-cell transcriptomic data from Planarian Digiworm ( https://digiworm.wi.mit.edu/ ) indicates that some of the highest-expressing cells for both genes are found in the protonephridial cluster (cluster 29), further supporting a role in protonephridial ciliary function (Fincher et al., 2018). This observation provides a link to the edema phenotype observed in a subset of RNAi knockdown animals. The lineage diagrams (Fig 1E, F) also depict inferred differentiation trajectories, in which increased gene expression toward the distal tips reflects enrichment in terminally differentiated cell types. Because the formation of motile cilia is expected to occur late in differentiation, the localization of *ropn1l* and *tex9* expression toward terminal branches is consistent with their proposed roles in motile ciliogenesis. Across major cell categories including secretory, muscle, gut, and parenchymal populations both genes show comparatively low expression, whereas enrichment is observed in specific neurons, epidermis, protonephridia, and pharyngeal lineages, all of which include tissues known to harbor abundant motile cilia (Fincher et al., 2018).

Our results show that *Schmidtea mediterranea* is a valuable *in vivo* model for studying conserved ciliary genes. Since defects in motile cilia are linked to human disease, analyzing ROPN1L and TEX9 helps clarify shared molecular mechanisms across tissues and species. Although ROPN1L and TEX9 function through distinct molecular pathways, both genes are strongly associated with spermatogenesis and sperm flagellar structure, suggesting a shared role in motile microtubule-based systems (Sharma et al., 2014). We therefore presented these genes together in an exploration of their potential involvement in ciliary structure and function. In summary, our findings demonstrate that ROPN1L and TEX9 are required for the maintenance of normal ciliary length and motile function in multiciliated cells in planarians. Integration of single-cell expression analyses with RNAi-mediated knockdown phenotypes—including impaired gliding motility, shortened cilia, and posterior abdominal edema—provides compelling functional evidence that these genes are conserved components essential for proper motile cilia assembly and performance. These results extend prior proteomic and transcriptomic associations by establishing direct roles for ROPN1L and TEX9 in ciliary biology.

## Methods

**Planarian maintenance and feeding protocol**

Planarians ( *S. mediterranea* asexual strain ClW4) were maintained in a dark environment at room temperature in vessels containing water prepared as follows: 1.6mM NaCl, 1.0mM CaCl _2_ , 1.0mM MgSO _4_ , 0.1mM KCl, 1.2mM NaHCO _3_ (Cebrià & Newmark, 2005).&nbsp; Worms were fed once weekly with organic beef liver paste. After two hours of feeding, any remaining food was removed using plastic transfer pipettes, and a small volume of fresh planarian water was added to top off the vessel. A complete water change was performed every two days to remove accumulated excretory waste and maintain optimal water quality (Rompolas et al., 2013).

**RNAi plasmid cloning and feeding**

Following the methods established in our previous study (Gogoi et al., 2026), RNAi constructs were generated and administered in *Schmidtea mediterranea* with gene-specific modifications as described below. FASTA transcript sequences for *ropn1l* (dd_Smed_v6_4888_0_1) and *tex9* (dd_Smed_v6_7007_0_1) were obtained from the PlanMine database, and Benchling was used to design primers generating 200–800 bp fragments with plasmid-homologous overhangs compatible with the pPR-T4P vector. A 543 bp fragment of *ropn1l* and a 615 bp fragment of *tex9* were amplified from cDNA using gene-specific primers with homology arms indicated in capital letters and gene-specific regions in lowercase (primer sequences available under Reagents). Total RNA was isolated from homogenized animals, reverse transcribed to cDNA, and target fragments were amplified using either Q5 or Taq polymerase, verified by gel electrophoresis, purified, and quantified to calculate a 2:1 insert-to-backbone ratio. Inserts were cloned into pPR-T4P using Gibson Assembly for *ropn1l* and InFusion assembly for *tex9* . Assembled constructs were transformed into *E. coli* DH5α, screened by M13 PCR, purified, and sequence-verified by nanopore sequencing prior to RNAi food preparation. The *C. elegans* gene *unc-22* with no known homolog in the planarian genome was used as a negative control *neg.cont.(RNAi)* (Wagner, 2012). Verified plasmids were subsequently transformed into *E. coli* HT115 for dsRNA production, and induced cultures were mixed with homogenized liver for feeding. Following a one-week starvation period, animals were fed six times over two weeks (every 2-3 days). For each RNAi condition, three experimental dishes containing ten animals each were analyzed (n=30).

**&nbsp;** *Seven days after the final RNAi feeding, phenotypic assays were performed as follows:&nbsp;*

**&nbsp;Cilia Phenotype Analysis** :

Planarians were immobilized by incubation in a relaxant solution (1% HNO _3_ , 0.8325% formaldehyde, and 50&nbsp;mM MgSO _4_ ) for 5 minutes prior to mounting on glass slides. Specimens were imaged using differential interference contrast (DIC) optics on a Zeiss LSM 510 Meta confocal microscope with a tungsten halogen lamp using a Plan Apochromat 63x/1.4 oil immersion objective lens with pixel size of 0.07μm/px. Images were captured using a Teledyne Lumenera Infinity 3 camera with Infinity Analyze software. For each condition, five worms were analyzed. Images were captured from both the left and right sides of the head region, near the eyes, where cilia are more densely distributed. From each image, the lengths of 10 individual cilia were measured, resulting in 10 data points per image. Measurements were performed using Fiji/ImageJ software to calculate the mean cilia length for both control and RNAi-treated planarians.

&nbsp; **Gliding Assays** :

Planarian locomotion was recorded over a 20-second period using an Andonstar AD246S-M HDMI Digital Microscope. Videos were analyzed in Fiji ImageJ, where z-projection was applied to generate tracks of individual worms. The lengths of these tracks were measured to calculate average swimming speed (mm/s).&nbsp;

&nbsp; **Statistical Analysis:**

Gliding velocity and cilia length measurements were quantified using FIJI/ImageJ software. For gliding velocity analysis, 30 individual worms were analyzed per condition. For cilia length analysis, 5 worms were used per condition, and for each worm, images were acquired from both left and right sides of the animal. Ten cilia were measured per side, yielding a total of 100 measurements per condition, which were averaged to obtain a single mean value per worm for statistical analysis.

The normality of replicates was confirmed using the Shapiro–Wilk test. Both RNAi groups ( *ropn1l(RNAi)* and *tex9(RNAi)* ) were compared with the same negative control *neg.cont.(RNAi)* animals. Analyses were conducted using GraphPad Prism version 10.6.1. One-way ANOVA revealed significant differences among groups for both cilia length (df=4, p < 0.0001) and gliding velocity (df = 29, p < 0.0001), and post hoc analysis using Dunnett’s multiple comparisons test further confirmed that both *ropn1* l *(RNAi)* and *tex9(RNAi)* worms differed significantly from negative control animals ( *neg.cont.(RNAi)* ) in both gliding speed and length of cilia (p < 0.0001 for all comparisons).

## Reagents

**Table:** 

**Category**	**Reagents**	**Use/Notes**	**Source of Reagent**
**Plasmids & Vectors**	pPRt4p plasmid	Expression vector backbone for cloning & RNAi experiments	Gifted by Jason Pellettieri
**Bacterial Strains**	HT115&nbsp; *E. coli*	RNAi feeding strain (RNase III deficient, used in dsRNA expression)	Gifted by Jason Pellettieri
&nbsp;	NEB 5-alpha&nbsp; *E. coli*	High efficiency cloning strain for plasmid propagation	New England Biolabs
**Primers**	*ropn1l* -fwd	CATTACCATCCCGaagctgccattcgtacccagcc	Invitrogen
&nbsp;	*ropn1l* -rev	CCAATTCTACCCGtgcttggttgaacgaggccacc	Invitrogen
&nbsp;	*tex9* -fwd	CATTACCATCCCGtccagcaaattccaacacgttcca	Invitrogen
&nbsp;	*tex9* -rev	CCAATTCTACCCGgctttctgacgctctagccttctgc	Invitrogen

## Data Availability

Description: 20 sec swim video of neg.cont (RNAi) worms. Resource Type: Audiovisual. DOI: https://doi.org/10.22002/z5vja-gfv92 Description: 20 sec swim video of ropn1l(RNAi) worms. Resource Type: Audiovisual. DOI: https://doi.org/10.22002/5m8tf-2aq36 Description: 20 sec swim video of tex9(RNAi) worms. Resource Type: Audiovisual. DOI: https://doi.org/10.22002/f41a4-hgf75 Description: edema observations. Resource Type: Image. DOI: https://doi.org/10.22002/s8625-b8x92
